# Remarks upon the Possibility of Urinous Vomiting, with Cases

**Published:** 1814-05

**Authors:** 


*For the Medical and Physical Journal.*

### *Remarks upon the Possibility of Urinous Vomiting, with Cases.*

IT is repugnant to my feelings to obtrude on the public an account of either my mental or bodily infirmities; but having read this morning the letter of Mr. Pulley on the case of Ann Fooks, inserted in p. 481 of the Medical and Physical Journal for December, and as it appears to me written in a spirit unfavorable to philosophical discussion, though my sun is declining too fast to permit me to seek any occasion for controversy, and though I am equally a stranger to Dr. Yeats as to Messrs. Gibbon and Pulley, I, as it were, feel myself constrained to state the following particulars, which incline me, notwithstanding all Mr. Pulley's sagacious and satirical strictures, to believe in the verity of the cases of urinary vomiting, stated and published by Drs. Senter and Yeats.

About five years ago I passed my grand climacteric, and the following winter I was attacked by a most severe and intractable cough, and in a few months I became subject to asthmatic paroxysms. The following year these complaints returned, and I then first experienced the pain and inconvenience attending *Ischuria.*

The cough, in defiance of all the usual remedies, became more frequent and harassing, and almost every morning, between half past two and three o'clock, roused me even if I were in a profound sleep, compelled me instantly to sit up in bed, and with a force as distressing as if "*soul and*

*body*

*body were riving*," continued for half an hour or more; then I had violent retchings to vomit, so loud and strong as to be heard in an upper story of my residence. These retchings would be repeated six to a dozen times or more, and I knew the cough would soon cease when I perceived a urinous taste. The two or three last vomitory exacerbations, generally brought up from two tea to three dessert spoonsful of limpid fluid; sometimes indeed it seemed mixed with saliva, and in either case it had as urinous a flavor as can possibly be imagined; then the cough ceased, and *tired nature* refreshed herself with *balmy sleep.*

These saline-aqueous ejections did not take place unless I was at the time more or less troubled with *Ischuria*, a complaint I am seldom sensible of during the day; previously to, and during its nocturnal visitations, I have a hard swelling in the abdomen, occupying a space equal to the size of my hand, rather to the right of the *umbilicus*, extending to the inguinal region, which swelling of the *hypogastrium*, after the urine is discharged, is scarcely perceptible; in the mean time the body is in a state of preternatural heat, the chest is incapable of bearing the least pressure, and the scrotum bathed as it were with moisture; but this last circumstance does not invariably occur.

My appetite has not been impaired by these severe sufferings, nor has my "*fleshy*"* appearance altered in the least, a proof among a multitude of others, that "*emaciation*" is not invariably the consequence of diseased action. My ailments, except occasional difficulty of respiration, notwithstanding my increasing age, are a little abated; my cough is now more manageable, its periodical return in the morning, and the attendant retchings, have not lately distressed me so much as they have formerly done, and medicine, when I have recourse to it, affords me more certain relief.

The determined sceptics concerning the possibility of urinous vomiting, will be disposed rather to ridicule than respect the facts I have stated; for Mr. Gibbon's coadjutor (Mr. Pulley) is very prompt in applying his *caustics.* In his essay now before me, he endeavors to be "*very sharp*" on Dr. Yeats. He tells us, he "thinks seriously—he "feels the most indignant desire to reprobate the imposition— "and yet he cannot altogether bring his mind to write gravely "on the subject!!!" †—He gives very intelligible hints that Dr. Y. "possesses a thirst for novelty, which can never be al- "layed, and an appetite for the marvellous of insatiable vora-

* Vide Mr. P.'s letter.

† Med. and Phys. Journal, No. 178.

"city, catching reports and assertions as they fly."* Having previously informed us of his demonological reading, and hinted at his inability to "*discern infernal gentry*,"† Mr. Pulley proceeds to tender us a bill of exceptions to *Dr. Senter's* case of Lucy Foster, declaring his "*disbelief entirely of this case*." Now, to a person who like me is an entire stranger to the state of parties at Bedford, there seems in this letter "*so full of sound and fury—more than meets the ear*." Has neither the demon of prejudice, nor "*green-eyed jealousy*," nor public, nor parish politics, insinuated their fermenting leaven into this physical discussion? Be that as it may, I, who am among the least of those who have been long devotees to Esculapian studies, feel regret at Mr. P.'s unprovoked and sarcastic strictures—strictures such as can only be justly applied to wilful stupidity, or detected imposture—strictures written in such a spirit, that, in my humble opinion, have a tendency to provoke the physician, if unfortunately he is a man of an irritable fibre, to relinquish his pen and appeal to his pistols.

At one of the consultations in which I had the honor a few years since to meet the late Dr. Samuel Foart Simmons, he presented me with two volumes of his "Collection of Medical Facts." The 13th and last article in the sixth volume,‡ consists of Dr. Senter's case of Lucy Foster. However "triumphantly" Mr. Pulley may insinuate that Dr. Yeats brought forward that case, it appears to me that the doctor could have done himself and the argument ampler justice, by introducing to the notice of his friends and the public, the additions subjoined to that case by the learned editor of the "Collection" just mentioned; who, far from discovering any flaws in the statement of the American physician, added other cases, tending to strengthen our belief of it.

Dr. Simmons thus writes: "There are many instances, Dr. Senter observes, in medical books, of sudden and partially increased actions of the vessels of the human body; but he candidly acknowledges, that his reading has furnished him with no fact similar to the extraordinary one which is the subject of the paper before us. There are however two

---

* The Drummer, or Haunted House, mayhap, where the Butler tells the Gardener—"Why a *Spirit* is such a *little*—*little* thing, that "I have heard a man, who is a great *scholar*, say, that he'll dance "ye a Lancashire hornpipe upon the point of a needle."

† Alas, I blame you not, for you are mortal—
And mortal eyes cannot endure the Devil.——*Shakespeare.*

‡ Published by Johnson, St. Paul's Church-yard, 1795.

cases which exhibit a striking analogy to those of Dr. Senter's patient; and, although they may have been overlooked, or perhaps disregarded, on a supposition of their improbability, they must now become extremely interesting by the tendency they have to corroborate the curious and extraordinary facts he has related. Both these cases we allude to occur in the History of the Academy of Sciences at Paris, and are as follows.

Case I.—" M. Maraldi has communicated to the Academy the following case, from a letter addressed to him by M. Marangoni, physician at Mantua:

" A nun of the order of St. Francis, in the convent of St. Joseph, at Mantua, aged thirty-five years, of a thin and delicate habit of body, and who had long been subject to hysterical complaints, was attacked with pains, spasms, and swelling of the abdomen, to which succeeded a violent and alarming suppression of urine. Soon after she felt a pain which she described as ascending from the lower part of the abdomen to the stomach, and she vomited a fluid which without any difficulty was known to be urine. This vomiting continued forty days, during which the patient voided no urine by the usual channel, unless the surgeon drew it off with a catheter, and even then the quantity scarcely amounted to an ounce a day. At the end of forty days the urine spontaneously resumed its natural course, and in a day or two the patient found herself perfectly recovered; but the vomiting of urine returned, and at the end of twenty days the patient complained of a very acute pain about the region of the pubis. Her surgeon was desirous of relieving her by means of the catheter, but there was such a contraction of the urethra, that he found it impossible to introduce his probe into the bladder. The vomiting of urine has continued, and what is remarkable, there is no appearance of food mixed with it, even when the vomiting takes place soon after her meals. When M. Marangoni wrote his account, the patient had been in this state thirty-two days.

" This singular complaint would lead one to think there is an immediate though hitherto an undiscovered communication between the stomach and urinary bladder; but M. Marangoni, and the celebrated Lancisi, are of a different opinion; they both of them think that in cases of this kind a suppression of urine takes place in the kidneys; that is to say, that the kidneys cease to extract this fluid from the blood, and that in their stead the glands of the stomach perform this function."

Case II.—" M. Lemery," (a name famous in the annals of medicine) " is acquainted with a monk, who for about eight years years was subject to a periodical vomiting, the fits of which are as regular as those of a quartan ague. Five hours, or thereabouts, before the vomiting begins, he complains of violent pains in his kidneys. The vomiting continues at intervals four or five hours. What he vomits is of a dirty red color. It is almost entirely water, but has a strong urinous smell, and the patient has no doubt of its being really urine, as he eats but very little, and drinks more than the usual portion of a monk. He drinks only wine, the color of which agrees with that of the fluid he vomits. A few hours after the vomiting he finds himself well, and remains so till the next fit. He uses a great deal of exercise, without which he thinks he should suffer more. It is a known fact, that in nephritic pains, which are always occasioned by obstructions of the kidneys, the patients are subject to frequent vomiting, and what they bring up smells much of urine."—"See Histoire de l'Academie Royale des Sciences, années 1715 and 1722. Editor."

I am firmly convinced of the possibility of urinous vomiting; "but for the denial of the utter possibility of that symptom, I should not" (says Dr. Yeats, vide letter, Aug. 8, 1813,*) "have engaged in the discussion at all." In the case of Ann Fooks, it is possible that the doctor may have been misinformed or imposed on, respecting some *minor* particulars; he however writes like a gentleman, disposed to sacrifice selfish consideration at the altar of truth.

In fine, it would give me pleasure to hear that peace waved her olive over the contending parties at Bedford; and that, under her benign influence, those who belong to a liberal and beneficent profession, desisted from pointing the finger of ridicule at each other; and instead of disputing like fierce and intolerant polemics, proved themselves emulous to concentrate their attention to those pursuits that tend to lessen the various sufferings of humanity.

*Islington, Jan.* 14, 1814. MEDICUS.

---

*For the Medical and Physical Journal.*

### *Cases of Fever in Children treated by Leeches.*

I WAS called to the infant daughter of Mr. B. residing in Tottenham-court-road, who was attacked the day before with symptoms of fever, viz. hot skin, florid countenance, thirst, sour breath; the tongue, nevertheless, was of a na-

* Med. and Phys. Journal, No. 175, p. 198.

